# Sociodemographic inequalities in the uptake of prenatal HIV testing in Ethiopia: Systematic review and meta-analysis

**DOI:** 10.1371/journal.pone.0308422

**Published:** 2024-10-22

**Authors:** Melsew Setegn Alie, Yilkal Negesse, Desalegn Girma

**Affiliations:** 1 Department of Public Health, School of Public Health, College of Medicine and Health Science, Mizan-Tepi University, Mizan-Aman, Ethiopia; 2 Department of Public Health, College of Medicine and Health Science, Debre Markos University, Gojjam, Ethiopia; 3 Department of Midwifery, College of Medicine and Health Science, Mizan-Tepi University, Mizan-Aman, Ethiopia; Auckland University of Technology, NEW ZEALAND

## Abstract

**Background:**

In order to attain the ambitious 95-95-95 UNAIDS goals, HIV testing serves as the pivotal starting point and plays a crucial role in preventing, treating, and managing HIV. Equal access to HIV testing is crucial to stop the spread of the virus. Measuring healthcare disparities is vital for promoting fairness in health services and achieving global goals for HIV prevention and treatment. Ethiopia is actively engaged in efforts to achieve these goals and is dedicated to combating HIV/AIDS. To the best of our knowledge, no previous systematic review or meta-analysis has been conducted on sociodemographic inequalities in prenatal HIV testing in Ethiopia. Hence, this study aims to assess sociodemographic inequalities in prenatal HIV testing in Ethiopia.

**Methods:**

We conducted an extensive search across various databases, such as PubMed, Scopus, Google Scholar, and Embase, to collect articles and reports. The data we gathered was then exported to R software for further analysis. Our analysis involved performing a meta-analysis of proportions using a random effect model. To assess the variability among the included studies, we used statistical measures such as I2 statistics and the Cochran’s Q test. The results of the pooled prenatal HIV testing, along with its corresponding 95% confidence interval, were presented using a forest plot.

**Results:**

A comprehensive analysis of 20 research papers on prenatal HIV testing in Ethiopia revealed that the overall pooled prevalence of testing was 69% (95% CI [60.0–80.0]). Factors such as higher education, urban residence, income, a positive attitude towards testing, marriage, and discussions about testing during antenatal care (ANC) were found to positively influence testing rates.

**Conclusion:**

The prevalence of prenatal HIV testing among antenatal care attendees in Ethiopia falls short of the UNAIDS target. In order to enhance the testing rates, it is advised to implement targeted initiatives within Ethiopia’s public health programs. To ensure effectiveness, these initiatives should adopt a sustainable and customized approach that takes into account the specific needs and circumstances of women, particularly those who are economically disadvantaged. Ethiopia can make significant strides in enhancing the prenatal HIV testing landscape by effectively implementing policies and programs that prioritize the welfare of the less privileged.

**Limitations:**

As limitation this systematic review did not include longitudinal and qualitative studies that might have provided different results.

**Clinical trial registration:**

Trial registration in PROSPERO with ID: CRD42024550564.

## Introduction

By the end of 2022, there were 39 million people living with HIV, with 1.3 million new infections. Women and girls accounted for 46% of these new infections, and sub-Saharan Africa had 65% of people living with HIV [1, 2]. Despite the availability of Antiretroviral Therapy, there were still 630,000 AIDS-related deaths in 2022 [1], with 60% occurring in sub-Saharan Africa and 44% affecting women and girls [3, 4]. HIV has far-reaching consequences, impacting households, communities, development, and economic growth. Inequalities in access to prevention and treatment contribute to higher rates of HIV infections and AIDS-related deaths, rooted in societal inequality [5–7].

The Joint United Nations Programme on HIV/AIDS (UNAIDS) has implemented programs to address HIV/AIDS inequalities [7, 8], but despite efforts, inequalities persist. Studies show socioeconomic disparities in HIV testing uptake in Sub-Saharan Africa, including Ethiopia [9, 10]. Higher socioeconomic positions increase the likelihood of seeking HIV testing [10]. Women bear a disproportionate burden due to poverty, gender disparities, and limited healthcare access [10]. They also face increased HIV risk during pregnancy and breastfeeding. Alarming statistics reveal high HIV rates among children in SSA, including Ethiopia [11–13]. Urgent comprehensive efforts are needed to address socioeconomic and gender inequalities perpetuating the HIV epidemic in this region [14, 15]. Equal access to testing and related services is crucial for women’s well-being and quality of life [16].

HIV testing during pregnancy is a crucial step towards preventing transmission, providing treatment, and offering supportive services [16, 17]. It serves as an entry point to PMTCT and helps to achieve the 95-95-95 targets set by UNAIDS for 2030 [18–20]. By identifying HIV-positive mothers early, we can prevent mother-to-child transmission and ensure access to necessary care [19]. Mother-to-child transmission refers to the transmission of HIV from an HIV-positive mother to her child during pregnancy, labor, delivery, or breastfeeding [21]. Although progress has been made, there is still a need for further advancements to fully meet the UNAIDS targets [3]. The remarkable decline in new HIV infections among children in sub-Saharan Africa is a testament to the effectiveness of PMTCT services and improved access to antiretroviral therapy for pregnant and breastfeeding women with HIV [22–25].

Ethiopia bears a significant burden of HIV/AIDS in sub-Saharan Africa. The Ethiopian Demographic and Health Survey (EDHS) report indicates that there are around 70,000 individuals living with HIV in the country, with females accounting for 62% of the total population affected by the virus. The prevalence of HIV among reproductive age women is 1.2% [26–28]. Ethiopia has made progress in combating the spread of HIV by implementing universal screening of pregnant women since 2007 [29]. The country is committed to eliminating mother-to-child transmission of HIV by 2020 and achieving the 95-95-95 treatment targets set by the United Nations in 2016 [20]. However, the prevalence and testing status of HIV/AIDS vary across different regions of Ethiopia due to sociodemographic and economic factors [30–32]. In 2016, only 34% of pregnant women were tested for HIV during antenatal care visits at the national level [28]. These disparities highlight the need for policymakers and stakeholders to address sociodemographic and economic inequalities in order to prioritize specific healthcare needs and improve prenatal HIV testing services [14, 33–42].

Equal access of health care is advocated by universal health coverage and end-inequality end AIDS epidemic also the goal of UNAIDS [43, 44]. In addition to this, United Nations’ Sustainable Development Goals (SDGs) aimed to reduce inequality [45]. UNAIDS recommends that each country establish evidence-based actions to reduce inequalities and eliminate AIDS as a public health threat by 2030 [44, 46, 47]. Recognizing the importance of understanding healthcare utilization inequalities, policymakers, researchers, and public health practitioners in Ethiopia are prioritizing efforts to enhance health systems [48]. However, it is crucial to note that sociodemographic inequalities in prenatal HIV testing at the national level have not been assessed in Ethiopia. Therefore, the objective of this study is to determine the extent of socioeconomic inequality in the use of prenatal HIV test services among pregnant women and identify factors contributing to these.

## Materials and methods

### Research questions

To conduct a systematic review of the pooled prevalence of socioeconomic inequalities in perinatal HIV testing and associated factors in Ethiopia, the research question was structured using the PICO (S) format. The participants (P) were individuals who tested for HIV during ANC follow up. The intervention (I) was testing services in a health facility, and the comparison (C) of this systematic review and meta-analysis was among those non-tested pregnant women, while the outcome (O) was the incidence of socioeconomic inequality during prenatal HIV testing. The study design (S) was all observational studies (cross sectional, case control, and cohort) and longitudinal studies (randomized control trials). This approach enabled the identification of relevant keywords to construct search strategies for a comprehensive literature search. The two research questions were: What is the pooled prevalence of HIV testing among pregnant women in Ethiopia? And what are the socioeconomic inequalities that influence women to receive HIV testing services in Ethiopia?

### Protocol and registration

This study officially registered with the International Prospective Register of Systematic Reviews (PROSPERO). The PROSPERO registration number of this systematic review is CRD42024550564.

### Data sources and search strategy

This systematic review and meta-analyses were conducted in accordance with the Preferred Reporting Items for Systematic Reviews and Meta-analyses (PRISMA) reporting guideline [49]. We performed an extensive literature search using various electronic bibliographic databases such as PubMed/MEDLINE, HINARI, Web of Science/Scopus, EMBASE, Google Scholar, and African journals online. Additionally, we searched the online repository of universities in the country and cross-referenced the included studies to find any additional relevant articles. Our search terms encompassed a wide range of keywords related to our topic, such as ’pregnant’, ’women’, ’gravidity’, ’predictors’, ’factors’, ’determinants’, ’risk factors’, ’associated factors’, ’utilization’, ’test’, ’testing’, ’practice’, ’HIV’, ’AIDS’, ’HIV/AIDS’, ’human immunodeficiency virus’, ’acquired immunodeficiency syndrome’, and ’human immunodeficiency virus/acquired immunodeficiency syndrome’. We used Boolean operators (AND/OR) to combine these search terms effectively. For example, in PubMed, our search query looked like this: [(pregnant OR women OR gravidity) AND (PMTCT OR prevention of mother-to-child transmission OR mother-to-child transmission) AND (HIV OR human immunodeficiency virus OR AIDS OR acquired immunodeficiency syndrome OR HIV/AIDS OR human immunodeficiency virus/acquired immunodeficiency syndrome) AND (predictors OR factors OR determinants OR risk factors OR associated factors) AND (utilization OR test OR practice AND HIV OR AIDS) AND (Ethiopia)]. To manage the references, we utilized EndNote software version X8 to remove any duplicated references and for citation purposes. This comprehensive search strategy allowed us to gather a wide range of relevant articles and ensure the accuracy and reliability of our findings. We independently screened the titles, abstracts, and full texts of articles. In cases of disagreement regarding the inclusion of a full-text article, all authors participated in discussions to reach a consensus. To ensure transparency and adherence to a standardized approach, we developed and registered a review protocol with PROSPERO. We used Boolean operators such as “AND” and “OR” during database searching (S1 File).

### Inclusion criteria

This systematic review employed a comprehensive approach called PICOS (participants, intervention, comparison, outcome, and study design) to select relevant studies for inclusion. Any papers that did not meet these criteria were considered irrelevant and excluded from the review. The included studies encompassed a range of study designs and examined the determinants of prenatal HIV testing in Ethiopia. The review specifically focused on papers published in English until February 22, 2024, and included observational and longitudinal studies. To assess the quality of each article, the Newcastle-Ottawa quality assessment scale (NOS) tool was utilized, and all articles successfully passed the quality assessment.

### Exclusion criteria

We excluded certain types of studies related to prenatal HIV testing. Specifically, we excluded studies that primarily focus on communication, as well as reviews, commentaries and letters to editors. Additionally, we have excluded studies where the raw data was not able to be analyzed, and protocols that were specifically conducted in Ethiopia.

### Study data management

The screening process consisted of two stages: an initial assessment of the titles and abstracts, followed by a full-text screening. To ensure accuracy, two independent reviewers utilized the EndNote software to evaluate the potential relevance of each article for further review. The assessment was based on a predefined set of inclusion and exclusion criteria. In instances where there were discrepancies between the reviewers’ assessments, they were resolved through discussion and by seeking input from a third reviewer. To ensure transparency, electronic records were maintained for both the included and excluded studies, with clear explanations provided for any exclusions made.

### Quality assessment and risk of bias

To assess the risk of bias in the study, a quality assessment checklist for prevalence studies was employed. The checklist of the Newcastle-Ottawa quality assessment scale (NOS) consists of nine items that are crucial in evaluating the quality of a study [50]. The evaluation of the primary studies involved assessing several factors, including target population representativeness, sample size, response rate, data collection tool, study case definition, comparability, assessment of outcome, and statistical analysis. Each of these items is given a score ranging from zero to nine, with a higher score indicating a lower risk of bias.

To ensure a thorough assessment, three authors (MS Ali, YN, and DG) independently evaluated the quality of the studies. In cases where there were differences in scores among the authors, the mean score of the three authors was used to resolve any discrepancies. In order to maintain the reliability of the results, studies that were found to have a high risk of bias were excluded from the final analysis. This step was taken to ensure that only studies with a lower risk of bias were included in the analysis, thus increasing the validity of the findings. Finally, twenty studies that received a quality score of low risk were included in the final analysis (S2 File).

### Data extraction

The process of extracting data involved using a pre-designed Microsoft Excel template. The form underwent multiple rounds of testing and revision as necessary. To ensure accuracy, a standardized data extraction tool was used. Two authors, YN and DG, independently performed the extraction. In cases where there were disagreements between the data extractors, the principal author, MS Alie, facilitated discussions to resolve them. The extracted descriptive variables covered various aspects such as region, study design; study period, data collection method, sample size, number of prenatal HIV testing, response rate, and socioeconomic factors associated with prenatal HIV testing.

### Sensitivity analyses

We performed a comprehensive sensitivity analysis to evaluate the influence of individual studies on the overall estimation of prevalence. Each study was systematically excluded, and we carefully assessed the resulting impact on the estimate. Surprisingly, the removal of any single study did not have a significant effect on the pooled prevalence estimate. Additionally, none of the studies deviated beyond the lower and upper boundaries of the confidence interval. These findings suggest that the combined results of the studies remained robust and consistent, thereby reinforcing the reliability of the overall prevalence estimate.

### Data synthesis and analysis

The data obtained from individual articles underwent processing using Microsoft Excel 2013 and was subsequently exported to R software version 4.3.2 for further analysis. Our analysis focused on 20 individual studies with the aim of determining the overall prevalence of socioeconomic inequality among antenatal care (ANC) attendant women in Ethiopia. To achieve this, we conducted random-effects meta-analyses for socioeconomic status reported in two or more studies using R software. This allowed us to estimate the pooled prevalence, along with 95% confidence intervals (C.I.s). In order to compare cases and controls while adjusting for confounding factors, we utilized R software version 4.3.2 to estimate the odds ratios (O.R.s). A p-value of less than 0.05 was considered statistically significant. Given the expected heterogeneity, we employed a random-effects model and utilized I^2^ statistics to assess the level of heterogeneity. Specifically, I^2^ values of 25%, 50%, and 75% represented low, medium, and high heterogeneity, respectively. To assess publication bias, we examined the distribution of studies in a funnel plot. Deviation from a symmetrical funnel shape can indicate the presence of publication bias.

Additionally, we assessed the quality control of the study. The Cochran’s Q test was used to test for heterogeneity, with I^2^ statistics indicating low (25%), moderate (25%-50%), and high (>50%) heterogeneity. We also estimated pooled odds ratios for factors associated with prenatal HIV testing and considered statistical significance at a P-value of less than 0.05 of the I^2^. An explanatory variable was included if data was available from at least two of the studies. Furthermore, we performed subgroup analysis based on potential sources of heterogeneity. Additionally, we conducted leave-one-out sensitivity analysis to assess the influence of individual studies on the overall effect, which was presented in tables and figures. Random and fixed effect model were used during meta-analysis of sociodemographic factors.

## Results

### Overview of study identification and selection

**Fig 1:**
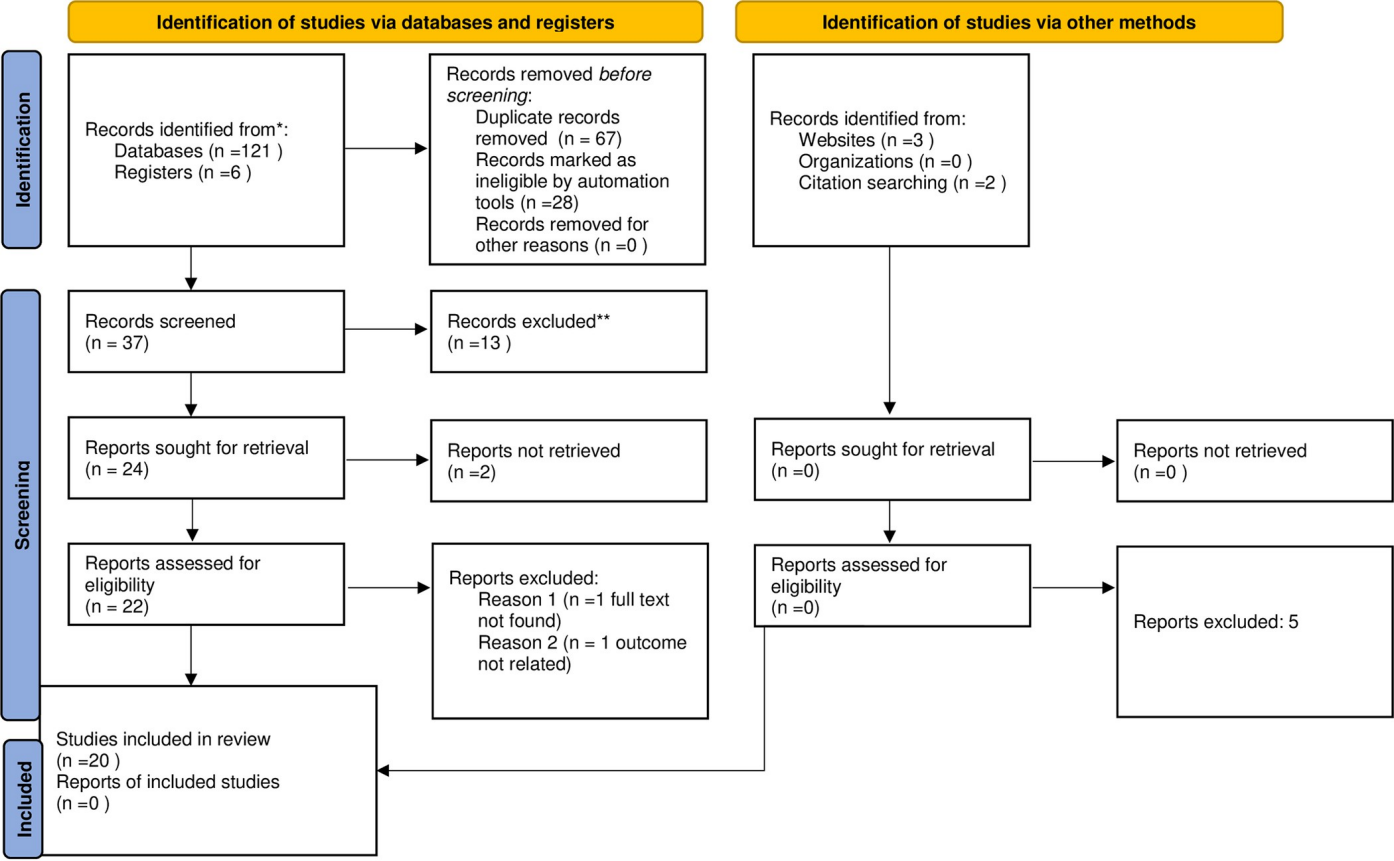
PRISMA flow chart shows study selection for systematic review and meta-analysis of socioeconomic inequalities for prenatal HIV testing in Ethiopia. **Reasons of retrieval protocol, review, unrelated and unclear outcome.

A comprehensive search was conducted using electronic databases and web-based sources, resulting in the identification of 132 records. To ensure accuracy, 67 duplicate records were removed using the EndNote application. In the second stage, we eliminated 28 additional records that did not meet the eligibility criteria. This left us with 37 records for further assessment. Upon closer examination, 13 articles were excluded, as they did not align with the study outcome, were review articles, or had unclear outcomes. Consequently, these articles were removed from the library. The remaining 24 articles underwent a thorough screening based on their full text. Two articles were excluded due to unrelated findings, leaving us with 22 articles for further consideration. During the full-text screening, it was discovered that two articles had a qualitative study design, and their full text was inaccessible for review. As a result, these articles were also excluded. Ultimately, 20 articles met all the criteria and were included in the systematic review and meta-analysis. For a visual representation of these results, please refer to Fig 1.

### Characteristics of the study

This analysis is based on a comprehensive review of 20 studies [30, 37–39, 51–65] conducted across all regions of Ethiopia. Out of the total number of studies, 50% (4 studies) were conducted in Amahara, 4 studies were carried out in Oromia, and five studies were conducted in the Southern Nation Nationality Peoples Region. Six studies did not provide information about their response rates. The sample sizes in these studies ranged from 216 to 2414 participants, resulting in a total of 10,248 participants from various backgrounds. The earliest survey was conducted in 2012, while the most recent one took place in 2023. It is important to note that the majority of these studies were found to have a low risk of bias. The response rate was reported in 14 articles, while 6 reports did not mention the response rate. For more detailed information on the included studies, please refer to Table 1 below.

**Table 1:** Descriptive presentation of the study included in systematic review and meta-analysis.

authors (year)	publication year	sample size	Number of prenatal HIV tested	Prevalence	response rate	Region
Deressa et al. (2014)	2014	843	791	94%	99.50%	Addis Ababa
Malaju et al. (2012)	2012	400	330	82.50%	97.60%	Amhara
Merga et al. (2016)	2016	374	325	86.90%	99.20%	Oromia
Zegeye et al. (2020)	2020	355	308	86.80%	98.60%	Amhara
Gebeyehu et al. (2020)	2020	340	234	68.80%	98.60%	SNNPR
Lema (2014)	2014	321	290	90.70%	100%	Oromia
Ejigu et al. (2017)	2017	2414	845	35%		Ethiopia
Dune et al. (2022)	2022	613	276	45(41.1–48.8)	97%	SNNPR
Gizaw et al. (2018)	2018	504	424	84.1(79.1,89.1)	98.60%	SNNPR
Gebremedhin et al. (2018)	2018	441	309	70.10%		Oromia
Kachero et al. (2021)	2021	798	508	63.7%(60–67%)	98.20%	Addis Ababa
Workagegn et al. (2015)	2015	308	160	51.80%	97.70%	Addis Ababa
Alemu et al. (2017)	2017	416	277	67.00%		Amhara
Facha et al. (2016)	2016	291	187	64.30%		SNNPR
Abtew et al. (2015)	2015	386	312	80.80%	97.00%	Benshangul Gumuz
Abajobir et al. (2013)	2013	232	223		96.10%	SNNPR
Akal et al. (2018)	2018	347	246	70.90%		Afar
Gebresillassie et al. (2019)	2019	364	298	81.70%	89.80%	Amhara
Kasaye et al. (2006)	2006	285	77	27.00%		Oromia
Yeshaneh et al. (2023)	2023	216	216	100%	100%	SNNPR

### Pooled prevalence prenatal HIV testing

**Fig 2:**
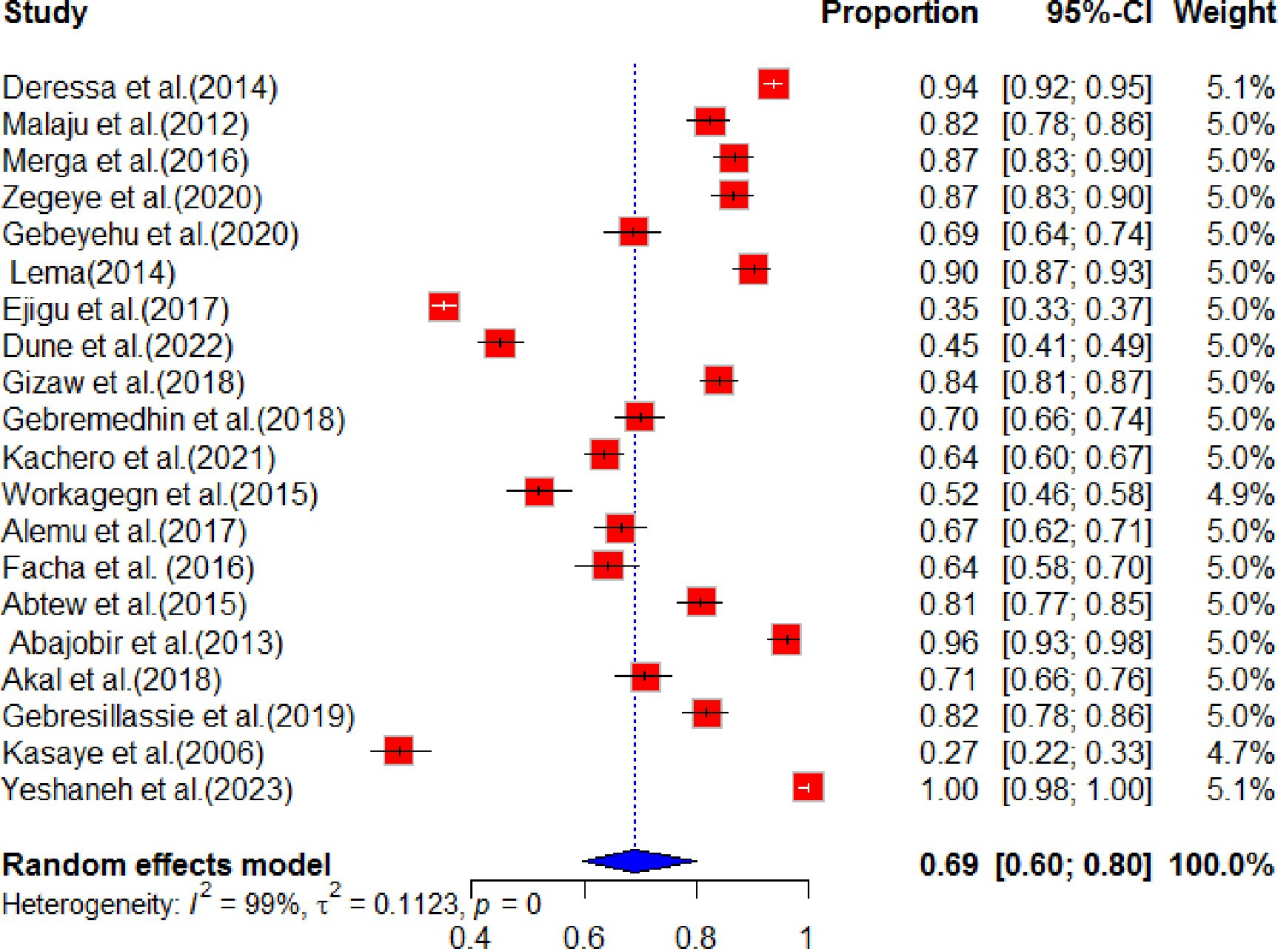
Forest plot of pooled prevalence of HIV testing HIV testing among pregnant women in Ethiopia, 2024.

A comprehensive analysis was conducted to determine the pooled prevalence of prenatal HIV testing among pregnant women. The findings revealed that in Ethiopia, the pooled prevalence of HIV testing among pregnant women were found to be 69% (95% CI [60.0–80.0]). This analysis also indicated a high level of heterogeneity, with a value of 99% and a p-value of 0.01. For a visual representation of the pooled analysis of HIV testing among pregnant women, please refer to Fig 2.

### Sub-group analysis of prevalence of prenatal HIV testing

A meta-analysis was conducted on prenatal HIV testing studies in Ethiopia, specifically focusing on the region and publication years. However, there was a shortage of studies in certain regions. The study revealed that in Addis Ababa, the capital city, the prevalence of HIV testing during pregnancy was 68.0% (95%CI [48.0–95.0]), with a high level of heterogeneity (I^2^ value = 99%). The highest prevalence of prenatal HIV testing was observed in the Amhara region, the second largest region in the country after Oromia. In the Amhara region, the pooled prevalence of prenatal HIV testing was 79% (95%CI [70.0–89.0]), with a heterogeneity value of 93% and a significant p-value of less than 0.01. The second highest prevalence observed in the Southern nation nationality people region, with a pooled prevalence of 74% (95%CI [58.0–94.0]).

Table 2 provides a more detailed sub-group analysis of the regions in the country. The analysis compared prenatal HIV testing before the final report of the Millennium Development Goal (MDG) and after the MDG or during the Sustainable Development Goal (SDG) period. Based on this sub-group analysis, it was found that pregnant women were tested for HIV more frequently before 2015 than after 2015. The pooled prevalence of prenatal HIV testing before 2015 was 72.0% (95%CI [45.0–100.0]), with a heterogeneity value of 98% (P value<0.01). In the pooled analysis of prenatal HIV testing after 2015, the prevalence was 69.0% (95%CI [59.0–80.0]). Furthermore, the sub-group analysis was done based on sample size to understand the effect of sample size on expected outcome and the accurate prediction of the studies. The included studies were categorized as having a sample size of less than 400 or greater than or equal to 400. The sub-group analysis revealed that studies with a sample size of less than or equal to 400 had a pooled prevalence of 73% (95%CI [62.0, 86.0]) for HIV testing among prenatal women in Ethiopia. For more detailed information of the results, please refer to Table 2 in the systematic review and meta-analysis.

**Table 2:** Sub-group prevalence of HIV prenatal testing among pregnant women in Ethiopia, 2024 (n = 20).

Variables	Characteristics	Included studies	Sample size	Prevalence(95%CI)	I^2^	P-value
Region	Addis Ababa	3	1,949	68(48–95)	99%	<0.01
	Oromia	4	1,421	62(36–100)	98%	<0.01
	Amhara	4	1,535	79(71–89)	93%	<0.01
	SNNPR	6	2,196	74(58–94)	99%	<0.01
	Other	3	3,147	59(35–97)	100%	<0.01
Sample size	≤400	14	4,635	73(62–86)	99%	<0.01
	>400	6	5,613	62(46–84)	100%	<0.01
Publication year	<2015	5	2,081	72(45–100)	98%	<0.01
	≥2015	15	8,167	68(59–79)	99%	0.01

Others = Afar, Benshangul Gumuz, country over all survey

### Risk of bias evaluation

The Newcastle-Ottawa Scale (NOS) was utilized to assess the quality and potential bias of the studies. This evaluation system takes into account three crucial factors: selection, comparability, and outcome. A maximum of 9 points can be assigned, with one author responsible for conducting the assessment and another author independently reviewing it to ensure accuracy. The overall score determines the level of bias risk. According to the evaluation using the NOS (S2 File), the studies included in this systematic review and meta-analysis demonstrated a low risk of bias.

### Meta-bias and sensitivity analysis

**Fig 3:**
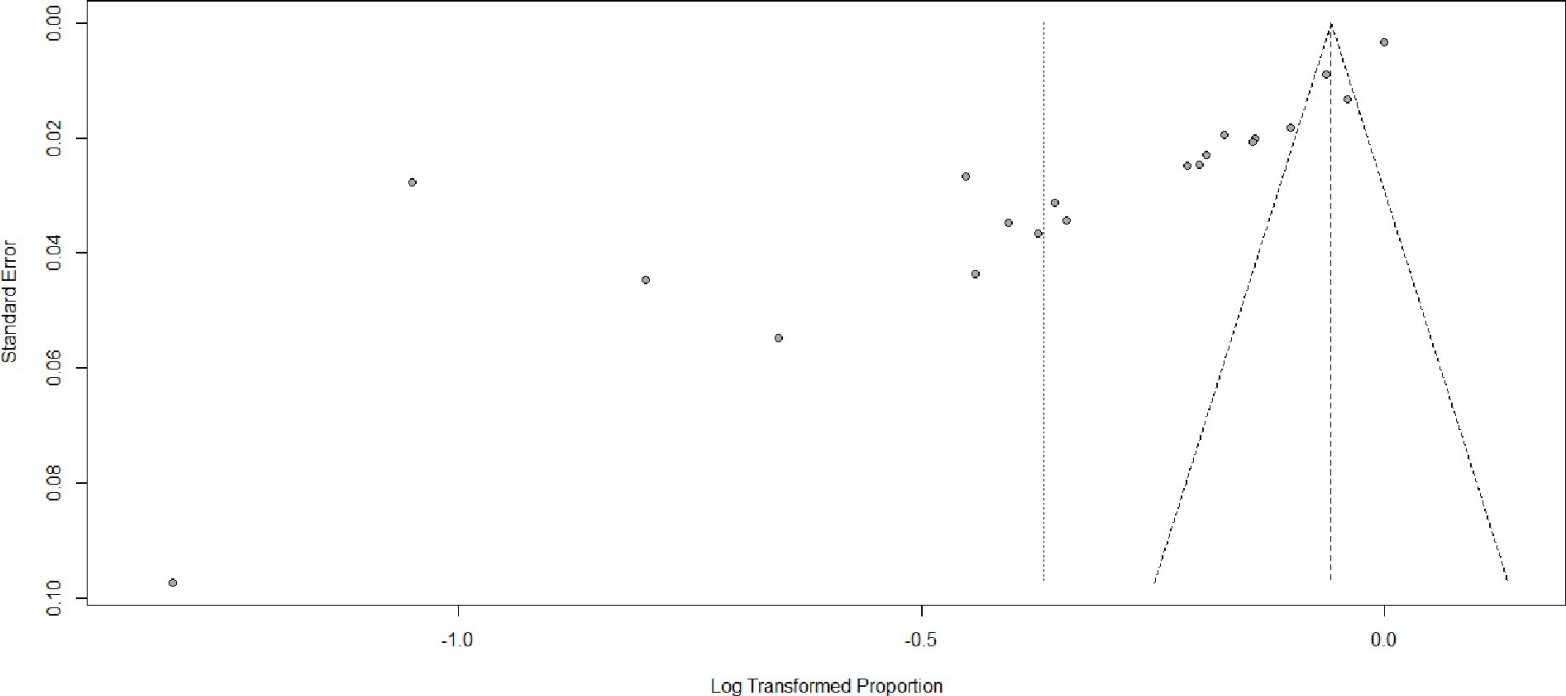
Funnel plot for systematic review and meta-analysis for prenatal HIV testing in Ethiopia, 2024.

**Fig 4:**
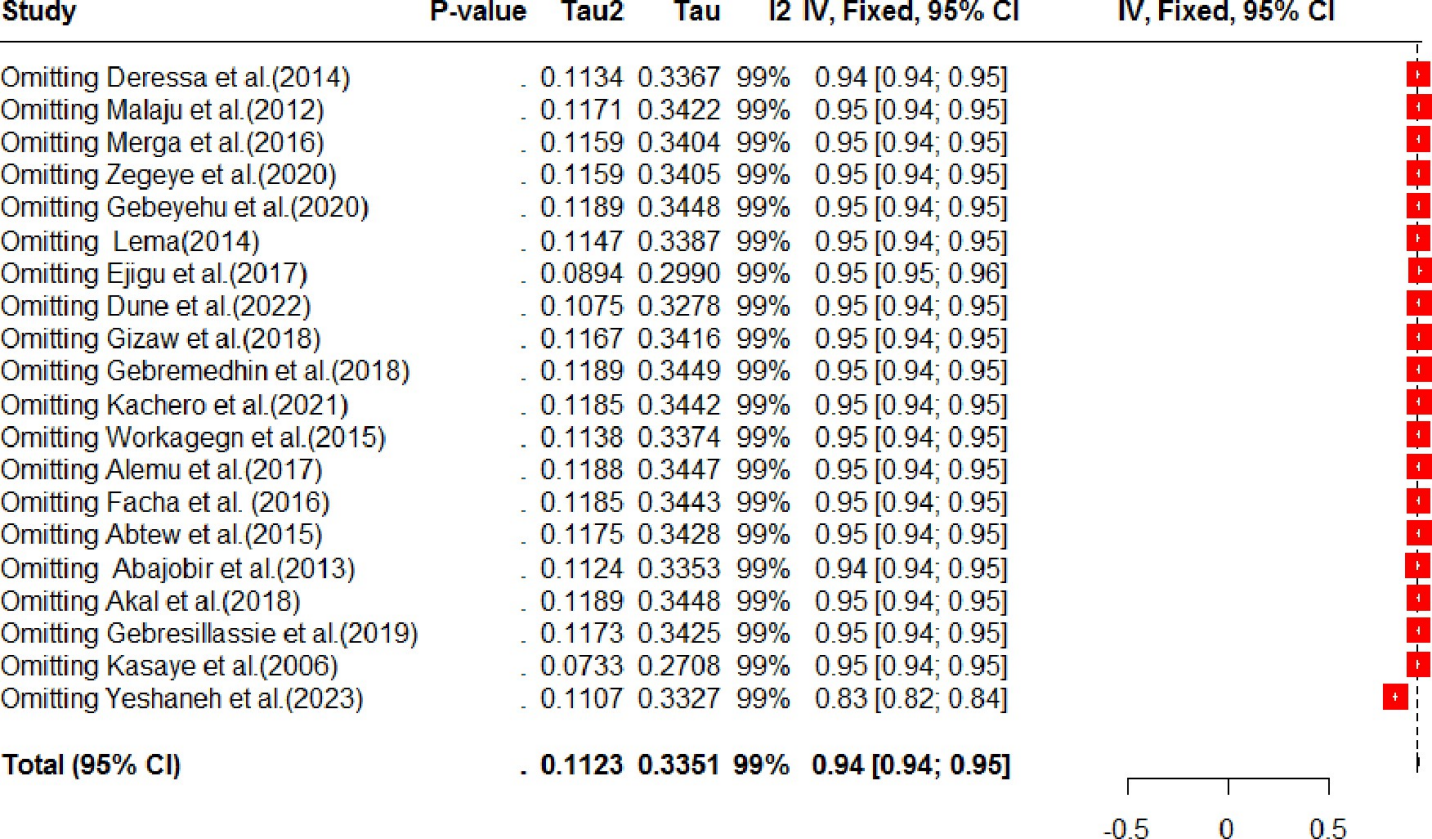
Sensitivity analysis for systematic review and meta-analysis on prenatal HIV testing in Ethiopia, 2024.

The presence of publication bias was assessed subjectively using a funnel plot, which revealed an asymmetrical distribution of effect estimates, suggesting the presence of publication bias (Fig 3). However, the Eggers test statistics indicated no significant publication bias (P = 0.21054). Additionally, a sensitivity analysis was conducted to examine the impact of individual studies on the overall pooled estimate of prenatal HIV testing in Ethiopia (Fig 4). The analysis found no evidence of any single study significantly influencing the overall pooled prevalence. Furthermore, a univariate meta-regression was performed, considering sample size and publication year as factors. However, neither of these factors was found to be statistically significant sources of heterogeneity (Table 3). It is worth noting that although the sample size was not a significant source of variability, there was a tendency for the prevalence of prenatal HIV testing to decrease as the sample size increased.

**Table 3:** Univariate meta-regression analysis to determine factors related to the heterogeneity of the prevalence of prenatal HIV testing in Ethiopia, 2024.

Heterogeneity source	Estimate	Se	Z value	P-value	CI	I^2^
Sample size	-0.0003	0.0001	-2.1772	0.0295	-0.0006–0.0000	99.64%
Publication year	0.0240	0.0199	1.2061	0.2278	-0.0150–0.0630	99.58%

### Association between sociodemographic characteristics and prenatal HIV testing

The association between sociodemographic characteristics and prenatal HIV testing was investigated in various primary studies, with different variables being considered. Specifically, the relationship between the educational status of women and prenatal HIV testing was analyzed by pooling data from multiple individual studies. Four articles [52–55] were examined to assess the association between secondary education and prenatal HIV testing, and all studies found a significant positive correlation. The meta-analysis revealed that women who attended secondary education were 3.68 times more likely to undergo HIV testing during the prenatal period compared to their illiterate counterparts (POR = 3.68, 95%CI [2.45–5.52]).To account for significant heterogeneity (I^2^ = 17%, p = 0.31) among the studies, a random effect meta-analysis model was employed. Furthermore, publication bias was evaluated using a funnel plot, which indicated the absence of bias. Two studies [30, 54] were included to examine the association between primary education and prenatal HIV testing in Ethiopia. However, the results were inconsistent, with one study showing a negative association and the other showing a positive association. Consequently, the meta-analysis concluded that there was no significant association between primary education and prenatal HIV testing. Notably, high heterogeneity was observed among the included studies (I^2^ = 90%, p<0.01). Finally, the association between higher education and prenatal HIV testing was investigated. The findings revealed a positive correlation, indicating that women with higher education were 3.34 times more likely to undergo HIV testing (POR = 3.34, 95%CI; [1.91–5.84]) compared to illiterate women (Table 4).

**Table 4:** Association between demographic factors and prenatal HIV testing in Ethiopia, 2024.

Variables	Included studies	Number of studies	Pooled AOR (95%CI)	Heterogeneity
Primary education	Gebeyehu et al. (2020)	2	0.79(0.19–3.30)	I^2^ = 90%, P value<0.01
	Ejigu et al.(2017)			
Secondary education	Lema (2014)	4	3.64(2.69–5.04)	I^2^ = 17%, P value = 0.31
	Ejigu et al. (2017)			
	Dune et al. (2022)			
	Alemu et al. (2017)			
Higher education	Ejigu et al. (2017)	2	3.34(1.91–5.84)	I^2^ = 0%, P value = 0.73
	Gebresillassie et al. (2019)			
Urban residence	Malaju et al. (2012)	4	3.16(2.14–4.65)	I^2^ = 0%, P value = 0.98
	Ejigu et al. (2017)			
	Alemu et al. (2017)			
	Abajobir et al. (2013)			
Age 25–34	Malaju et al. (2012)	3	2.20(0.42–11.59)	I^2^ = 90%, P value<0.01
	Merga et al. (2016)			
	Lema (2014)			
Being married	Lema (2014)	3	5.48(2.30–13.07)	I^2^ = 0%, P value = 0.95
	Gizaw et al. (2018)			
Wealthy women	Ejigu et al. (2017)	2	4.59(3.08–6.83)	I^2^ = 0%, P value = 0.68
	Alemu et al. (2017)			
	Gebresillassie et al. (2019)			
Gov’t employee	Merga et al. (2016)	2	0.36(0.01–14.11)	I^2^ = 94%, P value<0.01
	Abtew et al.(2015)			
Knowledgeable on PMTCT	Malaju et al.(2012)	6	2.37(1.68–3.35)	I^2^ = 49%, P value = 0.08
	Gebeyehu et al. (2020)			
	Gizaw et al. (2018)			
	Gebremedhin et al. (2018)			
	Kachero et al. (2021)			
	Alemu et al. (2017)			
Discussion on HIV testing during ANC	Merga et al. (2016)	2	6.26(4.21–9.29)	I^2^ = 0%, P value = 0.95
	Kachero et al. (2021)			
ANC follow up 2 and more	Malaju et al. (2012)	7	2.84(1.74–4.64)	I^2^ = 75%, P value<0.01
	Gebeyehu et al. (2020)			
	Lema (2014)			
	Dune et al. (2022)			
	Gizaw et al. (2018)			
	Gebremedhin et al. (2018)			
	Abajobir et al. (2013)			
Awareness of HIV testing	Gebeyehu et al. (2020)	2	2.14(1.62–2.83)	I^2^ = 0%, P value = 0.48
	Ejigu et al. (2017)			
Favorable attitude on HIV testing	Malaju et al. (2012)	2	3.72(1.49–9.29)	I^2^ = 63%, P value = 0.10
	Alemu et al. (2017)			

In addition, this study investigated the impact of residing in urban areas on prenatal HIV testing, by analyzing the findings of four research articles [39, 54–56]. The results of all four studies showed a positive and significant effect of urban residence on HIV testing during pregnancy. Through a meta-analysis, it was found that pregnant women residing in urban areas were 3.16 times more likely to undergo HIV testing compared to those living in rural areas (POR = 3.16, 95%CI: [2.14–4.65]). The fixed effect meta-analysis model was used due to low heterogeneity among the studies (I = 0%, p = 0.98) to estimate the pooled effect (Table 4). In this systematic review and meta-analysis, we conducted the association between age and prenatal HIV testing. Meta-analysis was conducted for three studies [39, 52, 60] conducted in the country, which showed significant association with prenatal HIV testing. According to the findings of this systematic review and meta-analysis, there appears to be no significant association between age and prenatal HIV testing in Ethiopia. Specifically, the study reported pooled odds ratio = 2.20 (95%CI: [0.42–11.59]) indicating that age did not have a significant effect on the likelihood of undergoing prenatal HIV testing. Therefore, it can be concluded that age is not a significant factor influencing prenatal HIV testing in Ethiopia, based on the results of this study.

Furthermore, the impact of knowledge on prevention of mother-to-child transmission (PMTCT) in relation to prenatal HIV testing was evaluated through the analysis of six research articles [30, 39, 55, 57–59]. The findings consistently showed a significant positive effect across all six studies. Specifically, pregnant women who possessed knowledge about PMTCT were 2.37 times more likely to undergo HIV testing during the prenatal period compared to those who lacked knowledge about PMTCT (POR = 2.37, 95% CI [1.68–3.35]). To determine the overall effect, a fixed-effect meta-analysis model was employed due to the low level of variability observed among the studies (I^2^ = 49%, p = 0.08).

The impact of attending antenatal care (ANC) two or more times on prenatal HIV testing was examined in a meta-analysis comprising seven articles [30, 39, 52, 53, 56, 57, 59]. The findings of the meta-analysis revealed a significant positive association between attending ANC two or more times and undergoing HIV testing during pregnancy. Specifically, women who attended ANC two or more times were 2.84 times more likely to be tested for HIV compared to their counterparts (POR = 2.84, 95%CI [1.74–4.64]). To account for the substantial heterogeneity observed (I^2^ = 75%, P-value<0.01), a random effects model was employed to estimate the combined effect of the variable.

In this meta-analysis, two articles [58, 60] were examined to assess the impact of discussions during antenatal care (ANC) on HIV testing among pregnant women. Both articles found a positive and significant association between discussing HIV during ANC follow-up and undergoing prenatal HIV testing. The results of the meta-analysis showed that women who engaged in discussions with their healthcare providers during ANC were 6.26 times more likely to practice prenatal HIV testing compared to those who did not discuss HIV during ANC follow-up(POR = 6.26, 95%CI [4.21–9.29]). To estimate the pooled effect of discussion during ANC on prenatal HIV testing, a fixed-effect model was used due to low heterogeneity (I^2^ = 0%, p-value = 0.95). This indicates that the studies included in the meta-analysis had similar findings, suggesting a consistent and reliable effect of discussing HIV during ANC on the likelihood of undergoing prenatal HIV testing.

Additionally, two articles [30, 54] assessed the impact of awareness on HIV testing among pregnant women, while three articles examined the relationship between wealth index and HIV testing. The findings revealed a positive association between awareness of HIV testing and prenatal testing. The meta-analysis demonstrated that women who were aware of HIV testing were 2.14 times more likely to undergo testing during pregnancy compared to those who were not aware (POR = 2.14, 95%CI; [1.62–2.83]). Similarly, the effect of wealth on HIV testing was evaluated in three articles [38, 54, 55], and a positive association between wealth and prenatal HIV testing was observed. The meta-analysis indicated that women from wealthier households were 4.59 times more likely to be tested for HIV during pregnancy (POR = 4.59, 95%CI; [3.08–6.83]). To estimate the pooled effects, a fixed effect model was used due to low heterogeneity.

Furthermore, two articles [52, 59] examined the relationship between marital status and prenatal HIV testing in Ethiopia. Both studies found a positive association between being married and undergoing prenatal HIV testing. To analyze the impact of marital status, a fixed-effect meta-analysis model was used due to low heterogeneity (I^2^ = 0%, P-value = 0.95). The meta-analysis revealed that married women were 5.48 times more likely to undergo prenatal HIV testing compared to single or never married women (POR = 5.48, 95% CI [2.30–13.07]). During the meta-analysis, the effects of age, favorable attitude, and being a government employee were also assessed. Three articles [39, 52, 60] examined the relationship between women’s age and prenatal HIV testing. Two studies [39, 52] had positive association with prenatal HIV testing while one study [60] had negative association with prenatal HIV testing. The pooled estimate from a random-effects meta-analysis model indicated that there was no significant association between age and prenatal HIV testing (POR = 2.20, 95% CI [0.42, 11.59]). Due to high heterogeneity random effect model was used to estimate the pooled effect of age on prenatal HIV testing (I^2^ = 90%, P value<0.01).

Finally, we investigated the effect of employment of prenatal HIV testing. Two articles [60, 62] examined the relationship between being a government employee and prenatal HIV testing. The combined results of a meta-analysis showed that there is no statistically significant association between these factors (POR = 0.36, 95%CI; [0.01–14.11]). A random effect meta-analysis model was used due to high heterogeneity (I^2^ = 94%, P value<0.01). Similarly, two articles [39, 55] explored the association between women’s favorable attitudes towards HIV testing and prenatal HIV testing. Both articles found a positive relationship between favorable attitudes and prenatal HIV testing. To account for high heterogeneity (I^2^ = 63%, P value = 0.10), a random effect meta-analysis model was employed. This meta-analysis revealed that women with a favorable attitude were 3.72 times more likely to be tested for HIV during the prenatal period compared to those with an unfavorable attitude (POR = 3.72, 95%CI; [1.49–9.29]) (Table 4). Due to the high heterogeneity, random effect meta-analysis was used to estimate the pooled effect of favorable attitudes.

## Discussion

This systematic review and meta-analysis synthesizes the sociodemographic inequalities in prenatal HIV testing among pregnant women in Ethiopia. The pooled estimate of prenatal HIV testing among pregnant women in Ethiopia was found to be 69% (95% CI [60.0–80.0]). This finding is comparable to studies conducted in Mozambique (75.4%) [66], East Africa (77.56%) [67], Africa (66.92%) [68], Gambia (75.4%) [69], and China (62.4%) [70]. However, it is lower than the findings from studies conducted in Kenya (83.9%) [71], Brazil (81.7%) [72], and the United States of America (75.2%) [73]. The variation in these findings may be attributed to several factors. Firstly, the wealth and resources available in each country can influence the implementation of healthcare programs. Countries with higher wealth, like Kenya, Brazil, and the USA, may have better healthcare delivery systems and more resources allocated to HIV testing programs. Secondly, differences in study design can also contribute to the variations observed. The methodology used, sample size, and sampling techniques may differ between studies, leading to different results. Lastly, the approach to healthcare and the availability of information can impact HIV testing rates. Countries like the USA, Brazil, and Kenya have better implementation and utilization of digital healthcare systems [74–76], as well as greater access to information about HIV testing through the internet [77–79]. This accessibility and digitalization of healthcare can contribute to higher screening rates. To achieve the UNAIDS targets by 2030, it is crucial to consider these factors and work towards digitalizing healthcare systems, increasing information accessibility, and improving healthcare delivery in resource-limited countries like Ethiopia. By doing so, we can enhance HIV screening rates and make progress towards the UNAIDS goals [80, 81]. Enhancing digital health in low-income countries holds immense potential in advancing the attainment of the three 95 targets and ultimately eliminating AIDS. This endeavor encompasses several promising factors that contribute to its success. By digitizing health records and implementing robust information management systems, healthcare providers can track HIV diagnoses, treatment initiation, and viral suppression rates in real-time. Such data-driven insights allow for targeted interventions and evidence-based decision-making to optimize HIV response strategies. This implies that multimodal, target-specific, and prioritized interventions with the help of digitalized health care highly improve the HIV testing status of women.

The findings of this systematic review and meta-analysis revealed higher rates of prenatal HIV testing compared to previous studies conducted in Zambia (46.9%) [82], Nigeria (54.4%) [69], and Congo (45.4%) [69]. The possible explanation for these differences lies in variations in study settings and methodologies. Additionally, country-level variations, cultural differences, and community perceptions towards HIV testing can also contribute to the variations observed. It is important to note that the presence of stigma and discrimination against HIV-positive individuals may also impact the HIV testing status of pregnant women in these countries. Therefore, implementing interventions that focus on socio-cultural factors, connecting HIV testing services, and working towards community-society-health integration, as well as providing support and care for HIV-positive individuals, may help improve testing rates and contribute to achieving the UNAIDS target.

The subgroup analysis revealed that studies conducted in regions such as the Afar and Benshangul Gumuz had the lowest prevalence of prenatal HIV testing compared to studies conducted in the SNNPR, Amhara, and Oromia regions. This difference could be attributed to variations in educational status, media exposure, and knowledge of HIV prevention methods. For example, the Ethiopian Demographic and Health Survey (EDHS) 2016 reported that respondents residing in SNNPR, Amhara, and Oromia had better comprehensive knowledge about HIV/AIDS compared to those living in Afar and Benshangul Gumuz regions [28]. This suggests that a lack of knowledge about HIV/AIDS might contribute to the lower rates of HIV testing in these regions. This implies that implementing awareness creation could improve the testing status of women.

This systematic review and meta-analysis identified various factors that influence HIV testing among women. These factors include secondary and higher education, urban residence, knowledge on prevention of mother-to-child transmission (PMTCT), attending antenatal care (ANC) two or more times, discussing HIV testing during ANC follow-up, awareness of HIV testing, wealth, marital status, and having a favorable attitude towards HIV testing. The findings suggest that women with secondary and higher education are more likely to undergo HIV testing compared to illiterate women. This finding is consistent with studies conducted in Eastern Africa [67], other East Africa study [18], Mozambique [66], Gambia [83], Rwanda [84], and United states of America [85]. The possible explanation for this is that education increases access to information and raises awareness about the benefits of HIV testing. Furthermore, educated women tend to have a better understanding of health information and are more likely to seek healthcare compared to those without formal education. This implies that the health care sector could place emphasis on counseling and information dissemination for women with no education for the achievement of the national HIV road map and the three 95% goals in 2030.

Furthermore, residence is one of the predictors of prenatal HIV testing. Pregnant women residing in urban areas were 3.16 times more likely to screen for HIV as compared with women residing in rural areas. This finding is comparable with the studies conducted in East Africa study [67], Mozambique [66], Nigeria [86], India [87], and the United States of America [85]. Similar studies conducted elsewhere [88–91] showed that women from urban areas were more likely to be tested for HIV as compared with rural women. The possible justification could be the implementation of almost similar policies and strategies for HIV testing. This implies that strengthening the rural health care program could improve HIV testing status.

The study found that women who had knowledge about prevention of mother-to-child transmission (PMTCT) were more likely to be tested for HIV during the prenatal period compared to those with poor knowledge of PMTCT. This finding is consistent with similar studies conducted in Kenya [71], India [87], and Rwanda [84]. One possible explanation for this finding is that individuals who have knowledge about PMTCT are more empowered to make informed decisions regarding their healthcare. Additionally, having knowledge about PMTCT increases trust in healthcare providers and improves healthcare utilization. Therefore, improving women’s knowledge about PMTCT can significantly enhance the utilization of HIV testing and contribute to the country’s efforts in HIV/AIDS prevention, aligning with the goals of UNAIDS. It is crucial to prioritize building comprehensive knowledge about PMTCT among women at the country level.

Pregnant women who attended antenatal care (ANC) appointments two or more times were more likely to undergo HIV testing compared to those who attended fewer appointments. This finding aligns with a similar study conducted in East Africa [67]. Regular ANC visits increase the opportunities for contact with healthcare providers, allowing pregnant women to receive more information about HIV testing during repeated interactions with these providers.

During the ANC follow-up, it was observed that women who had discussions about HIV testing were more likely to be tested for HIV, in comparison to women who never had such discussions. This finding aligns with a similar study conducted in Kenya [71], and Nigeria [86]. The possible explanation for this correlation is that exposure to information about HIV testing may contribute to an improvement in the overall testing rates within the country. Consequently, it is essential for prenatal care to include discussions on common and infectious diseases, such as HIV and syphilis, as these can have negative effects on pregnancy outcomes and maternal health. Pregnant women who are aware of the importance of HIV testing are more likely to undergo HIV testing compared to those who are not aware. This finding aligns with a study conducted in East Africa [18]. One possible explanation for this finding is that awareness increases individuals’ perception of their own risk and encourages them to seek better healthcare.

The study findings indicate that women from wealthier households were more likely to undergo HIV testing compared to individuals from poorer backgrounds. This pattern is consistent with similar studies conducted in Gambia [83], East Africa [18], 11 countries in East Africa [67], and Rwanda [84]. The higher socioeconomic status of these women appears to contribute to increased access to transportation and healthcare services, including private facilities. Additionally, the availability of media platforms such as TV and radio may play a role in reducing barriers to testing. Ultimately, these factors contribute to a higher likelihood of HIV testing among women from affluent households. This implies that lower socioeconomic women would be unable to afford the transportation costs compared with the richest individuals. So, expanding the HIV testing services to target lower income individuals and increasing health insurance to cover HIV testing costs are essential.

According to this study, it was found that married women were more likely to undergo HIV testing compared to single or never married women. This finding aligns with a study conducted in East Africa [67], and Kenya [71]. This suggests that being married increases women’s confidence in knowing their HIV status. Additionally, women in marital status may have fewer financial difficulties and face less social stigma when it comes to HIV testing and healthcare utilization. The attitude of the women is one of the factors identified in this systematic evidence synthesis. According to the study, women who had a positive attitude towards HIV testing were more likely to get tested compared to those with a negative attitude. This finding is consistent with previous studies conducted in other locations [39, 62, 92], which also found that a positive attitude towards HIV testing increases the likelihood of pregnant women getting tested. Healthcare decision-making is complex and influenced by various factors, such as attitudes and understanding levels. In this case, pregnant women who comprehend the significance of HIV testing for preventing mother-to-child transmission are more likely to get tested. This suggests that addressing individual cognitive and cultural perceptions could enhance the uptake of HIV testing in Ethiopia.

### Limitation

Although this research on prenatal HIV testing and sociodemographic inequalities in Ethiopia offers valuable evidence, it has a notable limitation. The study solely relied on cross-sectional data, which hinders the ability to establish a cause-and-effect relationship. This systematic review and meta-analysis included only papers published in English. To address this limitation, future researchers are encouraged to include longitudinal studies and qualitative research in their investigations. Furthermore, some studies still exhibited heterogeneity. In addition to the above limitation, some variables identified in a single study were not pooled in this systematic review and meta-analysis. In addition, most of the studies included were self-reported, so there could be a risk of social desirability bias.

## Conclusion

The overall pooled prenatal HIV testing among antenatal care attendants is low in Ethiopia as compared to the UNAIDS target [93]. The identified factors included higher education, being married, living in urban areas, having knowledge about prevention of mother-to-child transmission (PMTCT), attending at least two antenatal care (ANC) visits, awareness of HIV testing, belonging to a wealthier socioeconomic status, and having a favorable attitude towards testing. Therefore, policymakers and program designers could consider the sociodemographic factors for the effective implementation and success of HIV testing programs. Furthermore, emphasis could be given to the identified factors of prenatal HIV testing in Ethiopia. To improve the achievement of prenatal HIV testing in Ethiopia, policies and programs should take into account these determinants. Program interventions should specifically target women from poor households, rural areas, and those with unfavorable attitudes towards testing. It is crucial to engage rural women through education and skills development to enhance their knowledge and awareness of HIV and PMTCT, thus encouraging their participation in testing practices. Additionally, creating employment opportunities for economically disadvantaged women can improve their access to economic resources, empowering them to make informed decisions regarding HIV testing. Moreover, it is essential to enhance community HIV testing and index case testing approaches by focusing on women from impoverished households, rural areas, and those with negative attitudes towards prenatal HIV testing. It is crucial to provide robust counseling methods to effectively engage and support these individuals in accessing HIV testing services. In addition to this, the government of Ethiopia could implement a strong family focused HIV testing strategy and provide transportation initiatives for clients from low income households.

Furthermore, in order to achieve the goals of ending inequality and ending AIDS by 2030, it is crucial to take action and address the existing inequalities. Ethiopia, along with many other countries worldwide, is facing significant inequalities caused by various factors. Therefore, it is imperative for all regions and countries to implement comprehensive and multi-faceted strategies to combat these inequalities effectively. By doing so, we can work towards a more equitable and inclusive society for all [44, 94–96].

Education initiatives in communities should focus on providing accurate and factual information about HIV/AIDS while dispelling prevailing misconceptions. Emphasizing the availability of treatment to prevent HIV transmission to children born to HIV-positive women is also crucial. By implementing these strategies, Ethiopia can make significant progress in improving prenatal HIV testing rates and reducing the transmission of HIV from mother to child. We recommended the integration of prenatal services and HIV testing, considering women with low awareness of HIV, low use of ANC, and monitoring the effectiveness of the programs. The success of the Ethiopian national HIV road map intervention [97] relies heavily on addressing the identified inequalities. It is crucial to implement a comprehensive approach to HIV/AIDS programs. Pro-poor policy and program initiatives should prioritize a sustainable and customized approach that takes into account specific needs and circumstances. The findings will help policymakers develop targeted interventions to reduce the gap and improve access to HIV testing services during antenatal care.

## Supporting information

S1 Checklist PRISMA 2020 checklist. (DOCX)

S1 File Search terms summary result of sociodemographic inequality in prenatal HIV testing in Ethiopia. (DOCX)

S2 File The Newcastle-Ottawa quality assessment evaluation for cross-sectional study result for this systematic review and meta-analysis. (DOCX)

## Abbreviation and acronym

ANC: Antenatal care
ART: Antiretroviral treatment
EDHS: Ethiopian demographic and health sur
HIV/AIDS: human immunodeficiency virus/Acquired immune deficiency syndrome
PLHIV: People living with human immunodeficiency virus
PMTCT: prevention of mother to child transmission
POR: pooled odds ratio
SDG: sustainable development goal
UHC: universal health coverage
