# Supplementary material for: Sociodemographic inequalities in the uptake of prenatal HIV testing in Ethiopia: Systematic review and meta-analysis
Source: PLoS One. 2024 Oct 22;19(10):e0308422. doi: 10.1371/journal.pone.0308422 (PMC11495554; doi:10.1371/journal.pone.0308422)
Supplement: S1 File — (DOCX) [file pone.0308422.s002.docx]

S1 file: Search terms summary result of sociodemographic inequality in prenatal HIV testing in Ethiopia

| Database |  | Query | Items found |
| --- | --- | --- | --- |
| PubMed | #1 | ((((prenatal) AND (HIV)) AND (Testing)) AND (inequality)) AND (Ethiopia) | 2 |
|  | #2 | (inequality)) AND (prenatal)) AND (HIV)) AND (Testing)) AND (Ethiopia) | 2 |
|  | #3 | (((pregnant) AND (HIV)) AND (Testing)) AND (socidomographic enquality) | 4 |
|  | #4 | ((((HIV) AND (Testing)) AND (Prenatal)) AND (Inequality)) AND (Ethiopia) | 2 |
|  | #5 | : [(pregnant OR women OR gravidity) AND (PMTCT OR prevention of mother to child transmission OR mother to child transmission) AND (HIV OR human immunodeficiency virus OR AIDS OR acquired immunodeficiency syndrome OR HIV/AIDS OR human immunodeficiency virus/acquired immunodeficiency syndrome) AND (predictors OR factors OR determinants OR risk factors OR associated factors) AND (utilization OR test OR practice AND HIV OR AIDS) AND (Ethiopia)] | 72 |
|  | 6 | #1+#2+#3+#4+5 | 82 |
| HINARI |  | uptake of prenatal HIV testing in Ethiopia | 8 |
| Google Scholar |  | Sociodemographic inequalities in the uptake of prenatal HIV testing in Ethiopia | 26 |
| Embase |  | Inequalities in prenatal HIV testing and associated factors in Ethiopia | 4 |
| Scopus |  | Prenatal HIV testing inequality in Ethiopia | 6 |
| African journal online |  | Sociodemographic Inequlity in prenatal HIV testing In Ethiopia | 6 |
| Total |  |  | 132 |
