# Supplementary material for: Sociodemographic inequalities in the uptake of prenatal HIV testing in Ethiopia: Systematic review and meta-analysis
Source: PLoS One. 2024 Oct 22;19(10):e0308422. doi: 10.1371/journal.pone.0308422 (PMC11495554; doi:10.1371/journal.pone.0308422)
Supplement: S2 File — (DOCX) [file pone.0308422.s003.docx]

S2 file: the Newcastle-Ottawa quality assessment evaluation for cross-sectional study result for this systematic review and meta-analysis

| Quality assessment result of the study conducted on sociodemographic inequality in prenatal HIV testing In Ethiopia | | | | | | | | | | |
| --- | --- | --- | --- | --- | --- | --- | --- | --- | --- | --- |
| S/N | authors (year) | Representativeness of the cases | Sample size | Non-Response rate | Surveillance tool | Comparability | Assessment of the Outcome | Statistical test | Risk score | Risk rank |
|  |  |  |  |  |  |  |  |  |  |  |
| 1 | Deressa et al.(2014) | 1 | 1 | 1 | 2 | 1 | 1 | 1 | 8 | Low risk |
| 2 | Malaju et al.(2012) | 1 | 1 | 1 | 2 | 1 | 1 | 1 | 8 | Low risk |
| 3 | Merga et al.(2016) | 1 | 0 | 1 | 2 | 1 | 1 | 1 | 7 | Low risk |
| 4 | Zegeye et al.(2020) | 1 | 0 | 1 | 2 | 1 | 1 | 1 | 7 | Low risk |
| 5 | Gebeyehu et al.(2020) | 1 | 0 | 1 | 2 | 1 | 1 | 1 | 7 | Low risk |
| 6 | Lema(2014) | 1 | 0 | 1 | 2 | 1 | 1 | 1 | 7 | Low risk |
| 7 | Ejigu et al.(2017) | 1 | 1 | 1 | 2 | 1 | 1 | 1 | 8 | Low risk |
| 8 | Dune et al.(2022) | 1 | 1 | 1 | 2 | 1 | 1 | 1 | 8 | Low risk |
| 9 | Gizaw et al.(2018) | 1 | 1 | 1 | 2 | 1 | 1 | 1 | 8 | Low risk |
| 10 | Gebremedhin et al.(2018) | 1 | 1 | 1 | 2 | 1 | 1 | 1 | 8 | Low risk |
| 11 | Kachero et al.(2021) | 1 | 1 | 1 | 2 | 1 | 1 | 1 | 8 | Low risk |
| 12 | Workagegn et al.(2015) | 1 | 0 | 1 | 2 | 1 | 1 | 1 | 7 | Low risk |
| 13 | Alemu et al.(2017) | 1 | 1 | 1 | 2 | 1 | 1 | 1 | 8 | Low risk |
| 14 | Facha et al. (2016) | 1 | 0 | 1 | 2 | 1 | 1 | 1 | 7 | Low risk |
| 15 | Abtew et al.(2015) | 1 | 0 | 1 | 2 | 1 | 1 | 1 | 7 | Low risk |
| 16 | Abajobir et al.(2013) | 1 | 0 | 1 | 2 | 1 | 1 | 1 | 7 | Low risk |
| 17 | Akal et al.(2018) | 1 | 0 | 1 | 2 | 1 | 1 | 1 | 7 | Low risk |
| 18 | Gebresillassie et al.(2019) | 1 | 0 | 1 | 2 | 1 | 1 | 1 | 7 | Low risk |
| 19 | Kasaye et al.(2006) | 1 | 0 | 1 | 2 | 1 | 1 | 1 | 7 | Low risk |
| 20 | Yeshaneh et al.(2023) | 1 | 0 | 1 | 2 | 1 | 1 | 1 | 7 | Low risk |

Note: The scoring of the above items were as follow: Representativeness of the cases: 1=Truly and somewhat representative. 0=no description/selected demographic group user, Sample size:1=>400, 0=<400, Non-Response rate, 1=>95%, 0=<95%, surveillance tool: 2=Validated screening/surveillance tool. 1=Non-validated screening/surveillance tool, but the tool is available or described. 0=No description of the measurement tool. Comparability 1=The study investigates potential confounders, 0=The study does not investigate potential confounders. Assessment of the outcome: 2= Independent blind assessment and record linkage, 1=Self report, 0= No description. Statistical test: 1=The statistical test used to analyze the data is clearly described and appropriate.0=The statistical test is not appropriate, not described or incomplete.
